# The role of musical aspects of language in human cognition

**DOI:** 10.3389/fpsyg.2025.1505694

**Published:** 2025-03-21

**Authors:** Barbara Pastuszek-Lipińska

**Affiliations:** Department of Humanities, Academy of Humanities and Economics in Lodz, Łódź, Poland

**Keywords:** music, language, communication, speech therapy, human nervous system

## Abstract

This paper reviews musicology, linguistics, cognitive psychology, and neuroscience research on the importance of music in developing human speech and cognition. It cites research from several scientific fields on how the brain processes and reacts to melody, rhythm, harmony, loudness, dynamics and types of articulation and timbre. It also discusses musical concepts and prosodic features such as intonation, rhythm and stress related to linguistic terminology and summarises results of earlier research on how the two systems interact to strengthen or weaken an individual’s ability to function without nurturing stimulation. Music is an important preventive and therapeutic factor for human life. The author describes the interplay between music and language in the nervous system, improving or hindering communication and how it affects us personally and impacts societal mental health.

## 1 Introduction

The term prosody comes from the ancient Greek word προσῳδία¯, /prɔsoːˈdiaː/, meaning syllabic accent or song. Today, prosody deals with the linguistic features of the latent musical elements of spoken language. The study of the role of musical aspects of language in human cognition explores how prosodic features such as intonation, rhythm, and stress influence cognitive processes. It is important to introduce some theoretical concepts to understand the framework that integrates theories from musicology, linguistics, cognitive psychology and neuroscience, and the interplay between musical elements of speech and cognitive functions.

In the first few years of a child’s life, the brain undergoes intensive development, which impacts their understanding and processing of speech and language. As the development of our brains at birth is incomplete, we need to interact with the world to complete the process (Eagleman, 2020; McMullen and Saffran, 2004). Language development is then influenced by several factors such as genetic endowment, the conditions under which the child is born, and the environment in which the individual is raised. Research carried out at the turn of the 20th and 21st centuries has shown that another important element necessary for acquiring one’s first and then any subsequent language is the ability to process musical aspects of speech. In a recent paper by Brandt et al. (n.d.), the authors compare the acquisition of music and language to windows supporting the developing brain. These two systems have been found in some form in all known human cultures, and are acquired during normal childhood development. There is a significant overlap between them. Fiveash et al. (2021), on the other hand, argue that rhythm in speech and music is processed through common mechanisms and this may have implications for developmental speech and language disorders. Given that a child’s first contact with the world is based on the handling of sound and the transmission of emotions, through which the child signals their needs, reports discomfort or pain, or shows satisfaction and/or joy, an attempt was made to look at how so-called musical elements translate into speech and communication development. Interestingly, music is often cited as one of the most helpful interventions in cases of disorders or difficulties with speech production and is integrally connected with cognitive processes (Akanuma et al., 2016; Ashley and Timmers, 2017; Baker and Tamplin, 2006; Bitan et al., 2018; Christiner and Reiterer, 2018; Machado Sotomayor et al., 2021; Marchina et al., 2023; Merrett et al., 2014; Monroe et al., 2020; Slevc and Miyake, 2006). Juslin and Laukka (2003) emphasised the significance of controlling vocal patterns in speech production and singing, identifying a common thread in both language and music origins.

## 2 Music and language—two systems in one nervous system

Language and music are universal in human culture. Both systems use sequences of sounds organised temporally, either acoustically or as written language expressions organised into sequences of symbols. This study focused on the first form of expression. Temporal and rhythmic aspects are important properties of both music and language. These two systems involve organised acoustic signals used in interpersonal communication, and both involve complex cognitive and motor processes. These two systems and their relationships have attracted the interest of researchers from various disciplines. Although the similarities and differences between the two systems have attracted the attention of scientists and researchers from different disciplines for centuries (Drakoulaki et al., 2024), most of the analyses have been conducted over the last 40 years have attracted renewed interest from researchers as a result of new research methods becoming available to allow successive groups of researchers to start a new era of research on music, language, and the human nervous system.

New research methods and improvements in acoustics (including physiological acoustics, musical acoustics, auditory perception theory, as well as psychoacoustics), linguistics (especially phonetics, phonology, neurolinguistics, and psycholinguistics), musicology (music theory in its broadest sense, including aesthetics, psychology and music sociology), psychology (cognitive psychology, music psychology), but also auditory psychology, neuroscience, neurology, and neuropsychology of music.

The main motivation behind multidisciplinary research, especially its strong interdisciplinary aspect, can be expressed through three basic questions.

(1) What do language and music have in common, how are they related, and at what level can these relationships be observed?

The most obvious answer is that these two domains exist in all ancient and modern human societies. However, why both systems are present in all cultures remains a matter of debate. Although language is undoubtedly essential to human communication, the reasons for the universal nature of music remain unexplained.

The fact that music is ubiquitous in all cultures has been confirmed by archaeologists who have found evidence of musical activity dating from 40,000 to 80,000 years ago (Kunej and Turk, 2000; Peretz, 2006; Peretz, 2001). At the same time, some linguists have claimed that for biological reasons, the existence of music is completely useless (Pinker, 1997). It shows no signs of being designed to achieve a purpose such as living a long life, having grandchildren, or accurately perceiving and predicting the world. Compared to language, sight, social reasoning and physical ‘know-how,’ music could cease to exist for our species and the rest of our lives would remain virtually unchanged (Pinker, 1997). However, music exists and plays an important role in people’s lives. Mithen (2005) in his ‘Singing Neanderthals’ draws a picture of the common origins of music, language and body. This work provides an insightful and creative exploration of a crucial yet often overlooked aspect of history: how communication among our ancestors influenced their lives, maintained their communities, and ensured their survival. Therefore, is this really unnecessary for us? In section 3 consideration of this aspect of comparisons are presented showing common area of research but also gaps in the existing analyses.

(2) What are the reasons for studying phenomena that are so very different?

Language and music are complex systems, and working together in these domains can be challenging. Recent research has shown that music and language are more closely related than previously thought (Asano et al., 2021; Besson et al., 2007; Brown, 2017; Choi et al., 2024; Du and Zatorre, 2017; Honda et al., 2023; Hutka et al., 2015; Lehrdahl and Jackendoff, 1983; Jackendoff, 2009; Patel, 1998, 2003a, 2010, 2011, 2014; Patel et al., 2008; Schön et al., 2004; Toh et al., 2023; Wallin et al., 2000). Moreover, it seems that well-known cross-domain research can shed new light on the relationship between the two systems, as well as on each of them individually. Therefore, through a comparative analysis of music and language, we can obtain a more complex and coherent picture of the human mind than can be achieved by studying each domain separately (Choi et al., 2024; Du and Zatorre, 2017; Honda et al., 2023; Patel, 1998, 2003a, 2003b, 2010, 2011, 2014; Patel et al., 2008). In addition, the study of the development and evolution of language can benefit from considering music and language together (Bidelman et al., 2013; Brown, 2017; Christensen-Dalsgaard, 2004; Cross, 2001b; Cross, 2001a; Kraus and Chandrasekaran, 2010; Wallin et al., 2000). An attempt to answer this question have been provided in section 4.

(3) What are the limitations of interdisciplinary research as they were at the boundaries of the sciences?

When reviewing recent work and experiments to investigate the similarities and differences between music and language, it becomes clear that music and language share behavioural and functional characteristics. Recent neurophysiological experiments have shown common and overlapping brain substrates for music and language (Ogg and Slevc, 2019b; Overy, 2003; Patel, 1998, 2003a, 2003b, 2010, 2011, 2014; Patel et al., 2008; Slevc et al., 2016).

In the following sections the paper will provide an attempt to answer the overarching questions posited in the review.

## 3 What do language and music have in common, how are they related, and at what level can these relationships be observed?

To answer first question posited in the review it is worth nothing that the overlap have been observed in many research concerning the brain.

### 3.1 Neural overlap between language and music

The human brain is divided into two hemispheres. The right hemisphere has traditionally been identified with musical and holistic skills, while the left hemisphere has been identified with language processing and development. Some research, however, has changed the perception of this clear division. Experiments looking for musical centres have shown the absence of such centres, revealing that music perception and processing take place through the interaction of the two hemispheres. Moreover, the circuits active when processing music are also active when processing other sounds. A dynamic interaction between the two hemispheres has also been seen in other studies—e.g., Friederici and Alter (2004), which looked for lateralisation of auditory language functions. The authors proposed a dynamic dual pathway model to reflect a picture of the connections between the hemispheres active during auditory language comprehension. Friederici and Alter pointed out that none of these parts of the specialised networks are domain-specific, but that parts of these specialised networks are also involved in processing the temporal structure of sequences in the non-linguistic domain, namely music.

Several studies have convincingly confirmed the sharing of certain brain areas and circuits. These studies have raised controversy about Broca’s area, which after all, is still considered a specialised speech centre. However, several studies have provided evidence that this area is not specific to language but is also active during the processing of musical tasks (Friederici and Alter, 2004, p. 269; Koelsch, 2005; Patel, 2010, 2011, 2014; Patel et al., 1998a, 1998b, 2004, 2008). See also Dronkers et al., 2004 for more data concerning the role of the brain areas underlying language comprehension.

Furthermore, as suggested by researchers such as Rizzolatti and Arbib (1998), Rizzolatti et al. (1999), and Rossi et al. (2011), the motor properties of Broca’s area in humans are not exclusively related to speech. According to data obtained through Positron Emission Tomography (PET) and neuroimaging studies, Broca’s area can also become active during hand or arm movements (Rizzolatti and Arbib, 1998); as well as when people perform actions and view or hear other people’s actions (Bangert et al., 2006; Rizzolatti and Craighero, 2004; Gazzola et al., 2006; Keysers and Gazzola, 2006; Kohler et al., 2002; Rossi et al., 2011 and references cited herein).

Rizzolatti provided evidence for a *mirror system* or observation/performance matching system that enables gesture recognition and is common to humans and monkeys. According to Rizzolatti’s evidence, in humans, this system is in Broca’s area, which acts as a conduit between action and communication (Rizzolatti et al., 1999). Rizzolatti and Arbib claimed that the mirror system was crucial for the development of speech (Rizzolatti and Arbib, 1998). According to them, this is because ‘the development of the human lateral speech circuit is a consequence of the fact that even before the appearance of speech, the precursor of Broca’s area was endowed with a mechanism for recognising actions performed by others. This mechanism was a neuronal prerequisite for developing interpersonal communication and ultimately speech. Therefore, the authors describe language in a more general context than the one according to which speech is seen as its basis. These researchers also highlighted the claim of Donald (1991), who pointed out that the ability to imitate, a natural extension of action recognition, is central to human culture (such as dances, games and tribal rituals), and that the evolution of this ability was a necessary precursor to changes in the area of language (Iacoboni et al., 2001; Rizzolatti et al., 1999).

In the literature, we can also find evidence of separated pathways for processing language and music (Ogg et al., 2019). Ogg and Slevc, 2019a reported separable neural representations of sound sources allowing for discrimination of different timbres, speaker identity and musical timbre. Norman-Haignere et al. (2015) reported distinct cortical pathways for music and speech, and existence of a neural population for song in human auditory cortex (Norman-Haignere et al., 2022).

A recently growing body of research has provided evidence concerning the shared neural basis of music and language (Yu et al., 2017). These research provided results showing shared neurocognitive mechanisms (Asano et al., 2021), shared processing of both systems (Atherton et al., 2018). They also reported the joint prosodic origin of music and speech providing background to develop language (Brown, 2017; Patel et al., 1998b), evidence for a shared system based on the observed structural integration of language and music (Fedorenko et al., 2009; Patel, 2003a; Patel, 2003b; Patel, 2003c; Patel, 2012; Patel et al., 1998a, 2004, 2008). Studies by Asano et al. (2021) reported the existence of a shared neurocognitive mechanism; Anvari et al. (2002) the relationships between musical skills, and phonological processing, Patel (1998, 2003a), Patel et al. (1998a, 2004, 2008) reported common syntax processing, and shared pathways for speech encoding (Patel, 2011, 2014); Cohrdes et al. (2016) compared competences and skills in music and language on different level showing their close relationships. Jackendoff (2009) pointed out parallels and nonparallels between language and music showing their interdependencies.

To understand this interplay, it is worth looking at what happens in the human brain concerning the sound processing. To understand the power of music and language we need to understand the different elements involved in sound processing and why each plays such an important role. These two systems are active in the processing of sound, in the development of speech and, in the case of a brain-damaged person, in the rehabilitation process. The two systems in which each element that makes up the system plays an important role. This is because one element influences the others as they interact. In addition, a unifying element is necessary, i.e., tuning in to the person we are talking to, working with, and following them and their abilities. This would mean that to develop with sounds, a person additionally needs communication with other people (understood here as emotional attunement and synchronisation of movement and sound) (Fraisse, 1982; Gratier, 1999, 2003; Gratier and Magnier, 2012; Nummenmaa et al., 2012; Scheidt et al., 2021).

Researchers have pointed out that understanding the musical domain may increase our understanding of human cognition (Brandt et al., 2012; Rebuschat et al., 2011; Schlaug et al., 2005), outlined shared neurocognitive mechanisms (Asano et al., 2021), shared processing (Atherton et al., 2018; Sammler et al., 2013), and the joint prosodic origin of music and speech, providing background to develop language (Brown, 2017), evidence for a shared system based on the observed structural integration of language and music (Fedorenko et al., 2009). Zatorre et al. (2002) discussed the structure and function of the auditory cortex for music and speech providing inside into the functional organisation of the human auditory nervous system and the neural mechanisms responsible for processing music and speech, and evidenced that leftauditory cortical areas are better at temporal resolution and right auditory cortical areas at spectral resolution. Patel (2003c), on the other hand, proposed the shared syntactic integration resource hypothesis (SSIRH), outlining mechanisms that were subsequently discussed and confirmed by Slevc and Okada (2015).

Prior to this research, the school of nativists, including such as Noam Chomsky, which appeared in the 1950s, recognised that language was too complex a function to be learned and therefore needed special, innate cognitive mechanisms, a kind of instinct—a ‘mental organ of language’ (Chomsky, 1977).

For many years, researchers have been looking for mechanisms unique to the human brain that enable the use of language. Chomsky’s research began to focus on grammatical rules that allow sentences belonging to a language to be formed and understood. Meanwhile, the formal classification of language types in terms of grammatical rules and transformations is proving useful in the theory of artificial languages (mainly in computer science) but has still not been explained much in the case of natural languages.

Clearly, more attention needs to be paid to the processes taking place during the acquisition of linguistic competencies, which require favourable environmental factors to promote them. We now know that without stimulation and social relations, the innate linguistic potential may never be activated (Curtis, 1970; Fromkin et al., 1974) and the lack of adequate linguistic stimulation may prove to be an effective barrier to development (DeeDee, 2015; Shonkoff and Phillips, 2000). Language needs contact with other language users to form, which is only possible with interaction provided by caring and attentive caregivers (in psychology, this phenomenon is called emotional attunement; Huttenlocher et al., 2010) and is often mediated by musical interactions (Koelsch, 2020). Savage et al. (2020) even claim that music is a coevolved system for social bonding.

It is now known that the main structures involved in understanding and producing speech are located in the area around the angular cingulate gyrus, which is the association cortex for hearing, vision, and names; the area is involved in, for example, understanding metaphors or distinguishing the faces of individual people we meet (Kalbfleisch, 2004).

The brain is known to remain flexible throughout life and is capable of constructing new neuronal pathways to process language (Chai et al., 2016; Puderbaugh and Emmady, 2023).

In the left hemisphere of the brain, an area called Broca’s area plays a significant role in speech production, that is, an area that has neurons that deal with the functions of speech production and language comprehension. It is located in the anterior part of the left cerebral hemisphere, rostral to the primary motor cortex, and is essential for fluent and effective speech. Broca’s area appears to be essential for motor functions that deal with complex movements of the speech apparatus, that is, the tongue, lips, mouth, and vocal cords. It is noteworthy that speaking alone requires the use of about a hundred muscles, and at normal speech rates, it requires almost 150,000 neuromuscular events per second (Harandi et al., 2017). It is located in the frontal lobe and is responsible for syntax but also helps with planning, ordering, logical thinking, and rule acquisition. Damage to this area can cause motor aphasia (the inability to speak properly) and may also cause reading and writing disorders, even though the person still comprehends speech correctly.

Wernicke’s area is another area in the left hemisphere near the auditory cortex that is essential for speech comprehension, is Wernicke’s area. This centre is found at the intersection of sensory, auditory, visual, and tactile pathways where vocabulary is stored and neurons deal with comprehension. This is where speech sounds arrive after travelling through the ear and then via the auditory pathway to the central centres. This centre processes auditory information about the sound of words and enables the identification of words based on the sounds heard. Here, sounds are decoded based on a person’s earlier experience, transformed into words resulting in speech comprehension. Damage to Wernicke’s area, located in the temporal lobe, causes paraphasia (meaningless and ungrammatical speech), poor word choice and an inability to combine words correctly despite the subject being able to maintain normal rhythms and pronunciation.

In contrast, the process of broadcasting speech is as follows: first, a thought appears in the mind, which goes to Wernicke’s centre (Ardila et al., 2016; González et al., 2014). Next, information is sent via the arcuate bundle to the Broca’s centre and the primary ‘transmitting’ cortex. Movements of the muscles of the face, tongue, jaw, and larynx take place, thanks to which we speak the words we want to say. When we speak, we do so by using a certain timbre of voice; we utter sounds of a certain frequency, timbre, and volume, speak at a certain rate, and use certain utterances. All this is made possible by the workings of the brain and the signal we want to send.

The key factor in the initial stage of language development is the neurological development of the brain, this is the first and necessary condition for the later development of any language. The following subsections therefore make some observations about the language-specific areas of the human brain and how these brain areas can be linked to music processing.

Some researchers conducted comparative research on language, music, and action in cognitive neuroscience, and these keep finding evidence for both shared and non-shared components of cognitive systems (Asano and Boeckx, 2015; Asano et al., 2022; Fitch and Martins, 2014).

Given that humans have evolved species-specific capacities for both vocal imitation (speech and singing) and gestural imitation (speech and movement in general; Donald, 1991), a central question is whether language evolved initially as a system of vocalisation or gesture, as imitative mechanisms are critical to evolutionary accounts of language acquisition.

Adapting a domain-specific approach guides researchers to common structure-building mechanisms for language and music and provides data confirming shared structure-building mechanisms and syntax processing (Patel, 2003a; Patel, 1998; Sammler et al., 2013). Domain-general approaches suggest that perception and production rely on lower- or higher-level shared perceptual and cognitive processes, without the implication of a specific language-music processing mechanism (Drakoulaki et al., 2024).

A growing body of research results indicates positive transfer effect not only on language (speech), but also on movement after implementation of musical features such as rhythm and time-related musical features on movement (Burger et al., 2013; Fadiga et al., 2009). According to Watanabe et al. (2007) work with sounds means also work with movement as researchers observed an effect of early musical training on adult motor performance which suggests evidence of a sensitive period in motor learning. Chen et al. (2008b) evidenced that in our brain exists brain network for auditory-motor synchronisation which is modulated by rhythm complexity and musical training. Moreover, as Gentilucci and Dalla Volta (2008) showed in their study, that ‘spoken language and arm gestures are controlled by the same motor control system’.

The cited research findings may suggest the existence of a kind of loop that connects language, music, and movement (Gruhn, 2002; Fitch and Martins, 2014; Moreno-Núñez et al., 2021).

### 3.2 Music as a training of the brain

To continue the response to the question concerning the common areas and they interrelations it is worth nothing that these two domains can have impact on each other building the brain capacities.

The study of sounds in musicology studies the elements of a musical nature, which include melody, rhythm, harmony, agogics, loudness, articulation, and timbre. In addition, research conducted on the effects of sounds on the mood and well-being of listeners (Bradt et al., 2010; Granot et al., 2021; Kraus and Chandrasekaran, 2010; Trimble and Hesdorffer, 2017) explained how music is being a resource and may be used to obtain well-being goals. Fukui and Toyoshima (2008) showed that music may also ‘facilitate neurogenesis, regeneration and repair of neurons’ (p. 766–767). Another area of research is the relationship between the sounds of music and the activity of the hearing and listening brain (Kraus, 2021). The interested reader will find a detailed overview of research in Ozimek (2018) and Kraus (2021).

By the 20th century, scientists had already noted that the performance of music, the process of education and musical training supply complete training of the mind/brain (Weinberger, 1999a, 1999b) and since then the topic is still examined (Altenmüller and Schlaug, 2012; Loui et al., 2018). Such comprehensive training helps cell-to-cell communication by strengthening synapses, thus improving brain functions. As a result, increased creativity can be seen. For example, researchers who studied communication between jazz musicians during improvisation saw that this activity requires the musicians to be constantly curious about the auditory material, as improvisation is dependent on co-sensory (tuned) rhythmic coordination (Schögler, 1998; Stern, 1982), coordination and consonance (Setzler and Goldstone, 2020), synchronisation (Rasch, 1979) and social collaboration (Walton et al., 2018) between the musicians. This intuitive communication and coordination have been compared to the mother-infant relationship (Byers-Heinlein et al., 2020; Gratier, 1999, 2003; Gratier and Magnier, 2012; Papousek, 1996) during which young children listen to speech produced by a caregiver. Moreover according to Nguyen et al. (2020, 2021) neuronal synchronisation occurs.

Music psychologists have suggested that further research with musicians and an analysis of the skills they use in their work would improve understanding of the processes surrounding human communication (Deliège and Sloboda, 1997). Interestingly, curriculum vitaes of creative scientists and musicians active in several disciplines (Root-Bernstein, 2001), suggest that music played a vital role in this research.

These studies indicate that exposure to music seems to stimulate processes to improve brain circuits that are involved in the performance of various tasks (Chen et al., 2008b; Dalla Bella et al., 2017; Overy, 2003; Thaut et al., 2005; Weinberger, 1999a). Subsequent studies have shown that this includes linguistic tasks (Du and Zatorre, 2017; Kraus, 2021; Ludke, 2018; Ludke, 2020; Ludke et al., 2014; Nan et al., 2018; Patel, 1998, 2011, 2014; Patel et al., 2008; Wong et al., 2007) as language is a complex cognitive process that is essential not only for understanding, but also for thinking and functioning in the world.

Several areas of the auditory cortex are involved in decoding and representing different complex aspects of sounds. Information from the auditory cortex is then propagated to many other areas of the brain, notably the frontal lobe, which is involved in processes related to memory and interpretation; the orbitofrontal area, which is one of many regions involved in emotional evaluation; and the motor cortex, which works with the sensory-motor feedback circuits and controls movements needed to make music with an instrument (Quiroga-Martinez et al., 2024; Zatorre and McGill, 2005).

It is now known that musical skills require the combination and integration of several components, and it can be argued that the ability to play an instrument or sing requires a special gift. Music is, however, seen in virtually all cultures as a natural part of social life.

### 3.3 The brain and speech sounds—language development

Other studies answering the first question asked in the paper concern the language and interrelations of music and language point out the aspects relevant to sounds, and especially speech sounds.

It is clear from the description given above that processing sound necessitates hearing it. Sounds play a key role in our development, and appear in both language (speech) and music. Research in this area has understandably therefore focused on how speech is processed by the nervous system and how sound propagation occurs (Kraus and Chandrasekaran, 2010; Kraus, 2021; Patel, 2011, 2014).

When sound reaches the human brain, it triggers several processes that take place within it, playing a significant role in both healthy and damaged brains. The impact of the hearing brain on our functioning is enormous. It interacts with what we know about the world, with earlier experiences, emotions, what and how we think, our movements and all our other senses. Auditory neurons perform calculations with an accuracy of one-thousandth of a second. Hearing is also the fastest of our senses, which means that what reaches us through sounds can be a key record of who we become throughout our lives. The sounds that surround us from the beginning of our lives influence how our brains develop, and can stimulate or disrupt normal development (Blood et al., 1999; Kraus and Chandrasekaran, 2010).

Current levels of knowledge have already allowed us to trace how speech sounds are processed by the human brain (Kraus, 2021; Li et al., 2023). It is known that phonemic hearing is used to hear and understand speech, which makes it possible to distinguish speech sounds from one another, to divide words into syllables and to differentiate between words that sound alike, e.g., to distinguish between different phonemes and words differentiated by individual sounds. Sound waves cause the body to react and activate the amygdala—the part of the brain associated with memory and emotions (Blood et al., 1999).

Many studies point precisely to the importance of differentiation in development. This aspect seems particularly important in the case of language and music because here small deviations in sound can carry significant differences in the meaning conveyed, which can be a source of communication failure. Words that differ slightly in the sound of vowels or consonants trigger specific reactions in the areas of the human brain responsible for processing speech. False sound in music also have consequences at the neuronal level of the listener (Peretz et al., 2004; Schön et al., 2004).

It is known that all humans are born with a readiness to process all existing speech sounds (Papousek, 1996; Schögler, 1998). The development of a child’s speech from birth to age 7 can be divided into four periods: the melody, the word, the sentence, and specific infantile speech (Kaczmarek, 1977). Research on the speech acquisition process was conducted by Fernald et al. (1989) and Fernald and Morikawa (1993). Researchers found that the speech of all parents, regardless of culture, showed specific characteristics. Fernald’s research leaves no doubt that it is the higher frequency, elongated vowels and consonants articulated with excessive expressiveness that contribute to the stimulation of speech and language processing structures. Similar observations have been made with lullabies intuitively sung by mothers putting young children to sleep (Trehub and Gudmundsdottir, 2014; Trehub and Trainor, 1998).

Considering the findings of Fernald’s research cited above, it can be concluded that the musical aspects of speech directed to the child by caregivers during the first period of life play a vital role in the process of first language acquisition.

In the course of development, specialisation takes place. Based on the experience gained each day, more sounds, then syllables and finally, words and sentences are processed more correctly. With specialisation, the ability to hear sounds that are not found in the person’s environment decreases. The world and an increasing understanding of it begin to emerge from the world of sounds. Nevertheless, it all starts with sound and with the gradual feeling of differences in the sounds heard and differences concerning the context in which these sounds occur. This is of colossal importance for the development of the individual because global processing (right hemisphere), language functions gradually move to a specialised centre located in the left hemisphere. Research by Wong and his colleagues Wong et al. (2007) shows that after the critical period of language development, when we gradually become deaf to sounds with which we have had no contact, it is still possible to stimulate neuronal connections and create new pathways in the central nervous system. According to this study, as little as 5 h of contact with an unfamiliar sound creates new pathways in our brains—a neuronal recording that allows us to process that sound.

The content of our speech can be related to the past or stored memories, the present, which would be events occurring at the time of occurrence, and the future, which would be hypothetical or imagined events that our brain can generate. The amount of creativity is enormous. To generate speech, the parietal, occipital and temporal lobes, located in the posterior part of the cerebral hemispheres must be active in understanding currently occurring events or use memories. It is also believed that these areas help us to imagine future events. The left hemisphere participates in speech production and comprehension, while the right hemisphere is essential for communication as this area deals with figurative elements of speech such as understanding metaphors (Kalbfleisch, 2004).

And this is where another area common to music and language comes in, sounds. An aspect of both language and music that suggests similarities and a possible two-way interaction.

## 4 What are the reasons for studying phenomena that are so very different?

To answer this question it is worth focusing on their common aspects, especially the aspects of sounds in these two domains and the bilateral transfer between these two phenomena, the rhythm, harmony, articulation, tempo, timbre and finally potential applications in two-way transfer between the phenomena in question. All these qualities are present in both language and music and can be studied both separately and together.

### 4.1 Properties of sounds in music and language, i.e., musical aspects of speech

Musical aspects of speech refer to prosody called after Gibbon (2017) melodies and rhythm of speech, which concerns the melodic and temporal properties of speech that form the suprasegmental components^1^ of the phonology of a language and according to studies by Besson et al. (2007), Du and Zatorre (2017), Honda et al. (2023), Patel (1998, 2011, 2014), Tierney and Kraus (2014), and Toh et al. (2023), are susceptible to music influences (see also Zatorre, 2022).Gibbon describes melodies as ‘contours of the pitch values associated with syllables, words and whole utterances that contribute to rhythms whenever their pitch patterns alternate in similar time intervals, but also have additional properties of rising, falling or level pitch with their own functionalities. Rhythms and melodies which contribute to language structure and meaning constitute the domain of prosody’ (Gibbon, 2017, p. 1).

The processing issues of music and speech prosody have been studied by Schön and colleagues, who compared how musicians and non-musicians detect pitch contour violations in music and language (Schön et al., 2004). They found that subjects who had previously undergone intensive musical training were able to detect small frequency manipulations in both music and speech, while those without such training were unable to do so. Moreno and Besson (2005) conducted a set of event-related brain potential studies that examined the effects of musical training on pitch processing in children. Specifically, they provided the children with 8 weeks of music training and found that after this brief period, changes in pitch processing in language could be observed. Similar results were also reported by Magne et al. (2006), in an event-related potentials (ERP) study that examined the ability to detect pitch change in both music and speech, the authors showed that 3 to 4 years of extended musical training enabled children to perform better on this test, compared to those who had no such training. Jantzen et al. (2014) provided neurophysiological evidence confirming that musical training influences the recruitment of right hemispheric homologues for speech perception.

Pfordresher and Brown (2009) advocated the transfer from speech to music especially in tone-language speakers. ‘Results from [their] two studies suggest that individuals whose native language is a tone language, in which pitch contributes to word meaning, are better able to imitate (through singing) and perceptually discriminate musical pitch. These findings support the view that language acquisition fine-tunes the processing of critical auditory dimensions in the speech signal and that this fine-tuning can be carried over into nonlinguistic domains’ (Sammler, 2018; Chien et al., 2020). Chien et al. (2020) in turn demonstrated ‘cross-linguistic commonalities in the neural processing of intonation that overlaps with the phonological (but not semantic) processing of tone across Mandarin and German speakers. In contrast, semantic processing of tone was only observed in Mandarin speakers’ (p. 1853).

These studies confirmed the bidirectional transfer effect between music and language, as well as the existence of a common pitch processing mechanism in language and music. This transfer has been also observed in other studies such as further studies by Besson et al. (2007, 2011), Bidelman et al. (2011, 2013), Giuliano et al. (2011), and Moreno et al. (2009). In these studies researchers evidenced that a six-month period of musical training is enough to noticeably enhance behaviour and impact the development of neural processes, as indicated by specific brain wave patterns. These findings demonstrate the positive transfer from music to speech, underscoring the significance of musical training. Additionally, they highlight brain plasticity by showing that even short training periods can have substantial effects on the functional organisation of children’s brains. Moreno et al. (2009) reported for instance how musical training influences linguistic abilities in 8-year-old children providing more evidence for brain plasticity.

The transfer from music to language has been observed in several studies by Parbery-Clark et al. (2009), Strait et al. (2012), and Zendel and Alain (2012), and its impact on verbal memory confirmed studies by Chan et al. (1998), Ho et al. (2003), Parbery-Clark et al. (2009, 2011), Parbery-Clark et al., 2012, Strait et al. (2010), and Tierney et al. (2008).

Many studies have shown asymmetry for speech sounds and music, so different auditory information. Albouy et al. (2020) have been analysing distinct sensitivity to spectro-temporal modulation and found that this sensitivity supports brain asymmetry for speech and melody as they emerge from acoustical cues or from domain-specific neural networks. Their research provided one more evidence that the perception of speech and melodies depends on different types of acoustic information: temporal information for speech and spectral information for melodies (but see also Zatorre et al., 2002 and Zatorre, 2022). This asymmetry is reflected in the neural activity patterns in the left and right auditory regions, respectively. This finding highlights the specialised processing mechanisms in the brain for different types of auditory information. Similar results presented Sammler (2020), in her study concerning the split between speech and music she reported that the brain uses different neural pathways to process music and speech so, two types of auditory information. This research supports the idea that while there are shared resources in the brain for processing rhythm and pitch, there are also specialised networks that handle the unique aspects of speech and music.

However, some recent research contradicts these findings providing evidence ‘against the role of the language network in music processing, including the processing of music structure’ (Chen et al., 2021, pp. 34). Their results suggest that linking improvements in speech to music training may be a simplistic view as some other studies have provided a much more complicated picture. For a thorough review see Chen et al. (2021). and Honda et al. (2023).

### 4.2 Rhythm in music and language (speech)

Another aspects relate to the rhythmic organisation of music and language.

Rhythm appears to be a biological phenomenon that is central to our existence, and that involves the interaction of two distinct processes seen in both music and language: temporal grouping and rhythm induction. The first of these processes refers to how events are grouped or patterned in time. The second refers to the phenomenon of the beat or pulse that occurs with periodic temporal groupings.

Infants’ rhythmic movements in the first year of life are important predictors of later communicative development. Research showed that infants’ multimodal rhythmic movements increased the likelihood of adult responses. Adults offered several types of responses and closely observed the infant’s attention. This dynamic can support communicative development by promoting a framework of joint attention. In turn, this framework is essential if the nervous is to function correctly (Moreno-Núñez et al., 2021).

According to Gordon et al. (2015), musical rhythm discrimination explains individual differences in grammar skills in children. Rhythm provides people with synchronicity, harmony, or binding between internal and external elements of the environment, which is achieved through a system of so-called ‘internal clocks’ (Gibbon, 1977; Roach, 2001). These synchronisation and binding systems reflect the temporal organisation of the universe and environment (Chen et al., 2008a; Dalla Bella et al., 2017; Overy, 2003; Thaut et al., 2005). However, if the child is out of tune with the environment, several dysfunctions in language and speech development can occur (Levy et al., 2017). So how do we define rhythm in music and language (speech)? We know that both music and language use rhythm as the basic tempo of periodic events, so it provides the organisation of the elements being processed. Patel claims that rhythm in music and language benefits from the same resources and is very interdependent (Patel, 2003b).

The term speech rhythm refers to the perception of speech sounds (accented or unaccented). It is roughly equivalent to metre in music, which is defined as the regular repetition of accented and unaccented beats, which confirms the advocated co-dependency’s.

Plato claimed that ‘rhythm is the order of movement’ (Rudziński, 1987).

Following Rudzinski’s contribution to the area of rhythm, in music, the rhythm will be the orderly movement of sounds (gestures), because music and other fine arts are about movement given by man, foreseen by him, programmed, always according to the epoch, style, country, and individuality of the creator. The smallest movement having a beginning and an end is considered a model of movement (Rudziński, 1987). In language, on the other hand, and in speech in particular, it will be the ordered movement of sounds (gestures), because language is about movement given by man to generate a specific sound, foreseen by him, programmed, following his epoch, style, country, the intentions of the maker of the sound (author’s paraphrase).

According to Honing (2013b), rhythm can be considered to consist of several elements, such as rhythmic pattern, metre, tempo, and time. Most listeners can get these diverse types of information from an acoustic signal.

Gibbon (2017), on the other hand, argues that ‘rhythms are sequences of alternating values of some feature or features of speech (such as the intensity, duration or melody of syllables, words or phrases) at approximately equal intervals that play a role in the aesthetics and rhetoric of speech and vary somewhat across languages or language varieties under the influence of syllable, word, phrase, sentence, text and discourse structure.’ (Gibbon, 2017, p.1).

From the cited definitions, it is easy to deduce that rhythm serves to order a specific course in time and has a beginning and end (Feldman et al., 1999). Rhythm will therefore be a phenomenon that allows the sound material to be structured. This is where the phenomenon of the pulse comes in, which is the unit of structure in music and is referred to as the dominant level (bar) in the hierarchy of periods (metres). Thus, in music, the pulse serves as a means of linking rhythmic structure. Some also argue that the induction of rhythm is not a passive process but is a form of sensory-driven action that involves both sensory and motor components, suggesting a biological basis for rhythmicity (Feldman et al., 1999). Others, on the other hand, describe rhythm as a series of impulses, spaced more or less evenly in time, against which the timing of all musical events can be described (Dixon, 2001; Sierosławska, 2012).

At the end of the 20th century, an analysis of temporal phonology was tried and a dynamic approach to rhythm and language was developed. The rhythmicity of language was noted, and the fact that the timing and temporal structure of linguistic events at all levels (from the phonetic to the syntactic and semantic) are central to language processing since sentences and conversations are produced and interpreted in time (Cummins and Port, 1998; Port et al., 1995). In language, specifically in natural and functional phonology, the beat is treated as a primary rhythmic unit, which is realised by vocal figures and has no articulatory features. In this model, the minimal units of bit and non-bit are realised by vowel and consonant, respectively (Dziubalska-Kołaczyk, 1999, 2003).

Spoken language consists of sequences of speech sounds arranged in time; rhythm involves elements higher up the phonological hierarchy, and the domain of temporal patterns can include syllables, accent rates, prosodic phrases, sentences, and paragraphs. Temporal patterns can therefore vary significantly in length. This is consistent with the claim that temporal patterns, rather than absolute durations, are psychologically primary. Research on speech production also confirms the existence of hierarchical structures in phonology, which are derived from syllable structure, accent, and intonation (Jassem, 1962; Nakatani and Schaffer, 1978).

Port et al. (1995) analysed rhythmicity and strongly advocated a dynamic system that models the perception and production of linguistically controlled speech gestures. This phenomenon can be explained by examining the role of vowels in a sentence. Vowels, which occur at specific, predictable locations in a sentence, generate a rhythm that helps the listener to correctly perceive the message by focusing on these locations. Port and colleagues proposed ‘an oscillatory system that generates a rhythmic structure during speech production and [..] internally generates a similar perceptual rhythm when listening to speech’ (Port et al., 1995, p. 5). A similar model has appeared for metrical expectations during music listening. The experimental results of Port and colleagues suggest that rhythmicity can be directly correlated with measurable events in the acoustic signal. Rhythm is therefore an important aspect of spoken language and music. In both domains, it can be defined as a series of components that affect how the communicated information is organised in time, and several other parallels can be seen between time in speech and music. As already mentioned, rhythm is produced by the periodicity of a pattern, such as a syllable (which is a language-specific unit) or a motif (which is a music-specific unit).

In language, three main components of rhythm can be enumerated (Roach, 2001):

(1) The pattern of grouping/phrasing of words within utterances and pausing between utterances.
(2) The temporal pattern of syllables.
(3) The configurational pattern of accented and unaccented syllables.

In music, on the other hand, the following components of rhythm can be enumerated (Thaut et al., 2005):

(1) The grouping of sounds of different lengths into motifs, phrases, and sentences.
(2) The ordering of sound material in time; and then (if present).
(3) Periodicity on multiple time scales to create musical metre.

Rhythm also appears to be one of those elements of language that plays a key role in language acquisition since only a competent user who is fluent in the language can use it effectively and without boundaries and thus spontaneously communicate with others. In music, rhythm is one of the elements that directs the attention of the performer activates memory processes, and plays a key role in sequence perception and production as it organises phonic material. A growing body of evidence in recent literature shows interrelations between rhythm in music and language (Besson and Schön, 2012). However, Arvaniti (2009), postulated the need to reconsider our view of speech rhythm and focus less on timing, which should be examined separately from rhythm. She advocated adoption of a conception of rhythm going beyond timing and rhythmic types of languages and focus on grouping and patterns of prominence. This approach will enable connecting phonetic research with models of rhythm that are widely accepted in phonology and closer to the psychological understanding of rhythm. Consistent with Arvaniti (2009), Tierney and Kraus in their Precise Auditory Timing Hypothesis (PATH) provide evidence on how rhythmically-related motor entrainment in musical activities improves phonological awareness (Tierney and Kraus, 2014). The literature also provides some other recent empirical studies on the effect of rhythm vs. pitch training on phonological awareness (e.g., Patscheke et al., 2019).

In the musical domain, the results of the study by Thaut et al. (2005) revealed that music, through melodic rhythmic structures, enhances memory performance by mapping the temporal order in the material being learned (p. 252). The authors explained the effects induced by music: temporal synchronisation is a prerequisite for effective trace formation in memory. A musical pattern (song) for verbal learning induces cortical plasticity characterised by higher synchronisation in networks related to learning. Better synchronisation in learning-related networks may produce more stable neuronal traces for long-term memory. Increased synchrony in learning networks may also be the neuro-physiological basis for sustained music memory despite severe memory loss and improved access to verbal knowledge through music in neurological conditions such as dementia and Alzheimer’s disease. These data show that external rhythm as a temporal structure (in music) can drive the formation of internal rhythm in repetitive cortical networks for motor control and cognitive processes (Chen et al., 2008a; Dalla Bella et al., 2017; Overy, 2003; Thaut et al., 2005).

Given the observations made by Fraisse (1982), that people generally begin to synchronise their movements with a regular sequence of sounds early in life and quite naturally, the ‘strong psychological link between perception and production of rhythm’ and the ‘strong motor component in the psychological representation of rhythm’ have been highlighted. These phenomena have recently been investigated by many researchers in musicology, linguistics, cognitive psychology and neuroscience (Feldman et al., 1999; Levy et al., 2017; Macrae et al., 2008; Miles et al., 2009; Miles et al., 2010; Thaut et al., 2005).

### 4.3 Harmony

Next, phenomenon which have swimmingly important similar connotations is harmony. The word harmony comes from the Greek language. It is ‘the epitome of order and harmony,’ ‘conformity, mutual complementarity, or proper proportions,’ ‘harmony’, and ‘the manner of combining and building chords in a musical piece’.^2^

The cited definitions alone show that in both language and music, harmony serves to achieve a certain order, i.e., to combine successive elements of utterance or chords in a musical work in such a way as to achieve the effect wanted by the person uttering the words or creating the music (Ullman, 2006).

Undoubtedly, this element appears in both disciplines, and is what determines whether we find a given communication friendly and the sound pleasing to the ear (consonance) or whether we feel unease and a kind of ‘grating’ (dissonance).

Fedorenko et al. (2012) in their research focused on musical structure in the human brain, call the structure harmony. In linguistics, harmony serves to explain a type of assimilation, in which all vowels in a certain domain, usually the word, must agree in some phonological feature, such as roundness or backness (Fasold and Connor-Linton, 2006, p. 518).

### 4.4 Agogics (tempo)

Another important aspect concerning the common areas of language and music is tempo (agogics).

It is now known that how the stimuli leading to the acquisition of the first language and the formation of speech are presented is linked to the involvement of the right hemisphere of the brain. Research has shown that in processes related to the processing of auditory material, the auditory cortex of the right hemisphere is dominant in the encoding of syllable patterns (Abrams et al., 2008). In turn, it is the encoding of the temporal components of syllable processing that is important for correct speech comprehension (Fernald et al., 1989; Huttenlocher et al., 2010; Piazza et al., 2017).

The processes involved in processing slow (in the temporal sense) sound material, characterised by a slow pitch (3–5 Hz), take place mainly in the right hemisphere, while the same material is processed faster (20–50 Hz) in the left hemisphere (Piazza et al., 2017).

This means that the processing of sound material is related to the speed of this process. Recently, Ozaki et al. (2024) reported that globally, songs are slower, higher, and use more stable pitches than speech’ which may explain why some features of sounds are needed to train the human brain for speech processing.

### 4.5 Articulation

Articulation and pronunciation is another aspect that encourages the study of music and language together. When analysing the processes involved in articulation, i.e., how speech sounds are pronounced, it is useful to refer to articles describing research results on singing. Interesting information was provided by the results of two neuroimaging studies comparing brain areas active during music processing and language processes in the same people without musical training. It was seen that the performance of speech and singing tasks generated similar activity patterns but with a tendency to activate homologous centres in the opposite hemispheres of the brain, the left hemisphere for speech and the right hemisphere for singing (Zatorre et al., 2002). It has been shown that the right hemisphere is dominant during singing and the left hemisphere is dominant during speech production (Root-Bernstein, 2001).

Some studies have shown that bilateral activation is more pronounced during singing than during speech production and that the areas active during singing were not at the same time mirroring areas active during speech processing, which may indicate a more extensive network of connections activated during singing than previously supposed (Abrams et al., 2008, p. 3964). An analysis of the literature on the activity of individual centres during singing processing leads to the conclusion that singing activates areas of the primary motor cortex, such as the mouth region, as well as areas involved in laryngeal activity, that is, areas of phonation that are active during the stretching and relaxation of the vocal cords during sound production (Dronkers and Ogar, 2004).

The primary auditory cortex (i.e., the superior temporal gyrus, STG) is involved in vocalisation, for example, during the repetition of a single sound or the performance of more complex melodies. It has been recognised that other cortical areas are also involved in vocal production, such as the superior motor areas (SMA), anterior cingulate cortex (ACC) and anterior insular lobe. Higher motor areas are involved in higher motor control processes necessary for effective motor planning during the production of speech sequences (Dronkers, 1996, p. 160).

The anterior cingulate cortex is active during the initiation of speech-related vocalisation as well as during singing, while the anterior insula is associated with vocalisation processes, mainly articulation (Xu et al., 2004).

Despite the obvious differences between language and music, a significant overlap has been noted between the brain structures involved in processing singing and speech (Root-Bernstein, 2001).

These areas are responsible for auditory-motor integration (the inferior sensorimotor cortex and superior temporal cortex). This mechanism is crucial during vocalisation; for pitch monitoring, without it, it would not be possible to correct errors and fine-tune the pitch during singing. More specifically, the SPT area^3^ is activated during both silent humming and the production of inner speech. This area is considered a specific sensorimotor interface during speech production.

### 4.6 Timbre, or sound quality—spectral, or frequency properties of sound

The final aspect of both music and language is timbre, which refers to the quality of sound and the characteristics of the sounds produced when speaking, playing and singing.

Voice timbre is defined as a unique quality of sound. People can use different voice timbres when singing the same note. A silky voice sounds different from a throaty and a somewhat rough voice. The contrasts between different speech sounds are mainly based on timbre. Studies conducted worldwide and with people from all cultures show that mothers and people caring for young children speak differently to them than to adults.

In a study conducted with mothers talking to their children, researchers found a measurable specific vocal marker for each mother—an overall statistical profile of their voice timbre (Xu et al., 2004). Researchers have observed that speech directed at adults differs significantly from that directed at infants. In front of their children, mothers intuitively switch to a special communication mode known as ‘motherese’ or ‘baby talk’, an exaggerated and somewhat musical form of speech (Piazza et al., 2017).

Researchers have found voice timbre to be a feature that differentiates speech sounds depending on who the person is addressing. Timbre can support communication, build bonds, and pick out the voice of one’s caregiver from among the many voices. This is logical, as it is the specific caregiver who can guarantee the safety and development of the individual (Piazza et al., 2017, p. 3194). While this may sound surprising to adults, research has shown that it plays a significant role in language learning, engaging infants’ emotions and highlighting the structure of language to help children decipher the puzzle of syllables and sentences.

The researchers noted that all mothers indirectly used voice timbre and found that the change in the tone of voice was consistent across women from distinct cultures. Voice timbre is a consistent trait across all mothers, and is used as a switch between modes. It is important to remember that vocal descriptors such as raspy, gravelly, hoarse, nasal, and velvety refer to timbre rather than pitch. We used this property to distinguish between people, animals, and other sounds. Interestingly, in their paper, Mampe et al. (2009) also noted the influence of the native language of newborns on their cry melody.

### 4.7 Applications

Finaly while answering question number two concerning the reasons for studies these two disciplines together. It is worth asking what contributes to language competence and what stimulation supplies the best development of language and speech in the context of musical interactions.

The research review presented here provides the basis for the hypothesis that it is possible to strengthen language function through musical stimulation. However, only a few studies have documented the phenomena confirming the interdisciplinary transfer between music and language. These are research conducted by Wong et al. (2007), which showed how musical experience and the practice of music influence the processes responsible for language processing in the brain. Also, Du and Zatorre (2017) provided results confirming the positive effects of music training on speech perception through improved sensitivity to pitch and timing in that are required to understand spoken language (especially in noisy environments). This study constitutes one more study providing evidence of the shared neural mechanisms of music and speech and evidence concerning musical training and its effects on language skills. Another study was by Nan et al. (2018). This study shows how 6 months of piano training influenced pitch processing and speech perception. The training significantly improved the children’s ability to discriminate between different pitches and enhanced their speech perception, particularly in distinguishing consonants and lexical tones. This study provides additional evidence that musical training can enforce common sound processing mechanisms across domains, and benefit language processing Data confirming the enhancement of both perception and production of pitch-in-tone language speakers was presented by Pfordresher and Brown (2009). However, Ong et al. (2020) postulated that musicians show enhanced perception, but not production of native lexical tones, and Tao et al. (2021) that musicians may not show enhanced perception of native lexical tones in certain task settings, such as talker normalisation.

It turns out that there is a functional change under the influence of exposure to music, discernible in both behaviour and processing at the subcortical level. Wong et al. (2007, p. 422) investigated frequency processing and obtained results that showed better, that is, more precise, frequency processing by musicians. The study also revealed positive correlations between precision in frequency processing and the length and intensity of musical training and between the quality of frequency processing and the recognition and differentiation of syllables in Mandarin.

These results show that musical experience influences speech processing at the subcortical level and thus reflects long-term brainstem tuning to the experienced auditory stimulation, which would be thought to take place through neural design from the auditory cortex to subcortical centres. The researchers, point to the existence of the up-down connections already noted and suggest that the frequency processing noted for language is most likely improved through these connections.

The approach to auditory frequency processing postulated by Wong et al. (2007, p. 422) marks a new avenue for uncovering the functional role of poorly studied connections via descending subcortical pathways. This issue deserves more attention given that these pathways are highly susceptible to training and musical experience.

Confirmation of the role of stimulation (musical, linguistic and environmental) was recently made clear by Nayak et al. (2022) in their detailed review of language, musicality and environment Nayak et al. (2022) proposed the Musical Abilities, Pleiotropy, Language, and Environment (MAPLE) Framework for understanding musicality-language links across the lifespan. This detailed framework, based on a review of more than 70 behavioural and naturalistic studies, outlined research directions for future research on language development. The review underlies how neurobiological substrates may be strengthened ‘by genetic pleiotropy^4^ with musicality’ (p. 615) and highlights ‘that musicality is robustly associated with individual differences in a range of speech-language skills required for communication and development’. (p. 617).

Important research areas such as those focusing on individual differences are also emerging from several other studies. Tierney and Kraus (2014), for instance, offered a precise auditory timing hypothesis (PATH) showing how different approaches to musical training and ‘incorporating entrainment practice requires musicians to perceive the timing of acoustic events with a high degree of precision’ (p. 6). A gradual ‘increase in timing precision in the auditory system’s automatic representation of sound can be seen, which in turn leads to enhanced perception of the timing of speech sounds’ (p. 6) crucial for acquiring phonological skills, facilitating reading development. This model explains the key role of entrainment in musical practice and performance, which may be perceived as the area of individual differences.

Similar results have been reported by Patel in his papers and books (Patel, 2014; Patel, 2011; Patel, 2010; Patel, 2003b). For example, in his OPERA model (Patel, 2011, 2014), he suggested that neural coding of speech can benefit from musical training, but also suggested that several conditions must be met: overlap, precision, emotion, repetition and attention. Only in the presence of these conditions, can neural plasticity support speech communication processes. In line with Patel’s OPERA model, Choi (2020), and Choi with collaborators (Choi et al., 2024; Choi et al., 2023) investigated the role of musical instruments in music-to-language transfer in pitched and unpitched musicians and non-musicians (so also considering individual differences). They outlined ‘causal evidence for music-to-language transfer in lexical tone discrimination’ and, ‘the positive effect of music training on children’, which increased neuronal sensitivity to lexical tones. Interestingly, Choi also found that certain lexical tones may have specific acoustic features more relevant to musical experience suggesting that musical advantage was selective to certain lexical tones (2024, p. 361). Reported study data has shown musicians demonstrating not only an advantage in lexical tone discrimination and identification but also in non-native lexical tone sequence recall and word learning. These advantages were consistent with the lexical tone discrimination studies. Musicians outperformed nonmusicians in producing different sounds, including lexical tone. Interestingly although pitched and unpitched musicians outperformed the nonmusicians, pitched musicians showed a unique musical advantage in lexical tone discrimination and the largest musical advantage. In contrast, Burnham and colleagues (Burnham et al., 2015) investigated whether absolute pitch in the musical domain extends to the perception of lexical tones. The researchers found that people without musical training, who do not use tonal language, have impaired discrimination of pitch differences in lexical tones. This phenomenon indicates language-specific speech specialization. The researchers also noted that musical training can 'immunize or compensate for this specialization'. Musicians with absolute pitch (AP) 'have an additional advantage in accuracy', which the researchers interpreted as evidence that 'AP can be a general domain and not limited to a musical modality'. While the results of the Burnham et al. (2015) study show that 'musical training and absolute pitch ability are related to speech perception in a number of complex ways', they indicated that clarifying how and when this relationship emerges in development requires additional research.

A growing body of research also provides evidence on how musical interventions may be used in speech therapy to improve this process. A couple of studies reported the effectiveness of the Melodic Intonation Therapy (MIT) developed by Albert et al. (1973) and its results showed improved fluency (Helm-Estabrooks and Holland, 1998; Marchina et al., 2023; Monroe et al., 2020; Morrow-Odom and Swann, 2013), shorter words retrieval (Pastuszek-Lipińska et al., 2013). Reports by MIT have been published for over the last 50 years (Albert et al., 1973; Norton et al., 2009; Sparks et al., 1974) but not all of them succeeded in explaining which processes are the most important among a range of observed improvements. Merrett et al. (2014) gathered data on neurobiological, cognitive and emotional processes’ to better understand mechanisms generated by MIT.

Interesting input into the topic was provided by researchers examining how choral singing improves communication processes, including speech (Monroe et al., 2020). Baker and Tamplin (2006) published a manual concerning the application of music in neurorehabilitation processes also relevant to speech development and speech recovery. Herholz and Zatorre (2012) suggested that exploitation of ‘the effects of multimodality and reward that music might offer for plasticity, might be especially beneficial in elderly adults’ (p. 496) and after Wan and Schlaug (2010) claimed that ‘musical training might mitigate some effects of ageing in the brain’ (p.496). Schön and Tillmann (2015) provided evidence on short- and long-term rhythmic interventions and their views on language rehabilitation. For a thorough review of the emerging therapeutic applications using music, see Särkämö et al. (2016) and for the issues relevant to music-based interventions for mental illness, their opportunities and limitations, Golden et al. (2022).

Wolff et al. (2023) postulated in their paper that music engagement may be even seen as a source of cognitive reserve in different degradation illnesses, such as different kind of dementia. Brancatisano et al. (2020) in turn offered explanation on ‘why is music therapeutic for neurological disorders?’

The results give us more evidence of the strong connection between music, language and the brain.

## 5 What are the limitations of interdisciplinary research as they were at the boundaries of the sciences?

Usually, when we work at the intersection of different disciplines, we experience boundaries and limitations. Again, several topical issues need more attention and additional research, as some studies are opening up new avenues of research and others are still under-researched. As it was presented in the paper research at the intersection of music and language has made significant progress, yet several key issues remain underexplored. One such issue is the neurobiological mechanisms underlying the interaction between music and language processing. While already cited studies have shown that musical training can enhance language skills, the precise neural pathways and cognitive processes involved are not fully understood. Addressing this gap is crucial for developing targeted interventions in education and therapy.

Additional studies are therefore needed on the role of descending subcortical pathways in examining music-language interplay as suggested by Wong et al. (2007), on building cognitive reserves and mental health as postulated by Wolff et al. (2023) and on musicality-language links across the lifespan as postulated by Nayak et al. (2022).

Another area that requires further investigation is the cultural and social factors influencing the relationship between music and language. Research often focuses on Western musical traditions and languages, neglecting the rich diversity of musical and linguistic practices worldwide. Expanding the scope of research to include non-Western cultures can provide a more comprehensive understanding of how music and language interact across different societies.

Research concerning the mother–child interactions and musicians playing together in very good attunement are also underexplored.

Also all aspect of the impact of a composer’s language on the composed music and vice-versa should enhance more attention of researchers as also unexplored.

More attention should be also given to singing with words, as this aspect is still underrepresented in the research. Further research concerning the association between absolut pitch and the lexical tones occures in development is also needed.

Finally, it seems worthwhile to explore the impact of technology on music and language research, as this is now a growing field that definitely needs more attention. Advances in artificial intelligence and machine learning offer new tools for analysing complex data. Ethical issues and methodological challenges also need to be adequately addressed to ensure that these technologies are used responsibly in sensitive research on not only music and language, but also on cognitive development and maintenance of this aspect of everyone’s functioning.

## 6 Conclusion

This paper presents a brief overview of research on the musical aspects of speech at the developmental stage and a brief mention of speech therapy methods using music. The individual elements of a musical work, such as melody, rhythm, harmony, dynamics, agogics, articulation, and timbre are analysed, and shared areas appearing in speech that constitute a common area of interest for researchers from various fields of knowledge are introduced. This paper outlines the processes occurring in the human brain during sound processing, with particular emphasis on speech sounds.

The paper also presents an overview of research showing how the musical elements of speech help to enhance development and create healthy bonds and relationships between the child and caregivers, contributing to speech development through real communication manifested in attunement. It also shows how difficulties and speech-related problems can be addressed by incorporating music into the therapy.

## Footnotes

^1^suprasegmental, in phonetics, a speech feature such as stress, tone, or word juncture that accompanies or is added over consonants and vowels; these features are not limited to single sounds but often extend over syllables, words, or phrases (Brittanica).

^2^Definition retrieved from Słownik języka polskiego (1997).

^3^i.e. the dorsal cortex of the Sylvian sulcus at the parietal–temporal junction.

^4^Polygenic pleiotropy: When the same sets of genetic variants make contributions to two or more distinct complex traits, pointing to shared genetic architecture (Nayak et al., 2022, p. 617).
